# Analysis of Anastomotic Venous Factors in Traumatic Lower Extremity Injuries Reconstructed by Free Flap

**DOI:** 10.7759/cureus.20978

**Published:** 2022-01-05

**Authors:** Keisuke Shimbo, Rikuo Shinomiya, Toru Sunagawa, Yukako Okuhara, Nobuo Adachi

**Affiliations:** 1 Plastic and Reconstructive Surgery, Hiroshima Prefectural Hospital, Hiroshima, JPN; 2 Orthopedic Surgery, Graduate School of Biomedical and Health Sciences, Hiroshima University, Hiroshima, JPN

**Keywords:** recipient vein, two veins, end-to-side venous anastomoses, reconstruction, lower extremity

## Abstract

Background

Venous thrombosis has been shown to be the most frequent cause of free flap failure in traumatic lower extremity injuries. However, the roles of various anastomotic venous factors, including venous anastomosis (end-to-end (ETE) or end-to-side (ETS)), venous outflow (one vein or two veins), and recipient venous selection (deep or superficial vein), remain unclear. This retrospective study aims to investigate factors contributing to microvascular complications in patients with lower extremity Gustilo type IIIB/IIIC injuries reconstructed by free flap with a focus on the three abovementioned venous factors.

Methods

A total of 44 flap treatment outcomes of 41 patients with these injuries from 2015 to 2020 were assessed according to the three venous factors (type of anastomosis, venous outflow, and vein selection).

Results

The average patient age was 52 years, with the majority (75.6%) being male. Eight patients (18.2%) returned to the operating room due to venous thrombosis, and five patients (11.4%) experienced total flap failure. The following factors were suspected to have contributed to venous thrombosis: vein size mismatch (n = 2) and recipient vein insufficiency possibly due to post-traumatic vessel disease (PTVD) (n = 6). End-to-side (ETS) anastomoses showed lower venous thrombosis rates than end-to-end (ETE) anastomoses (6.3% versus 25%, p = 0.22), two-vein outflows had lower rates than one (8.3% versus 30%, p = 0.07), and deep veins had the lowest thrombosis rates (7.7%), whereas superficial veins had the highest (38.5%).

Conclusion

The key venous factors in preventing venous thrombosis include using as many two-vein ETS anastomoses as possible to deep recipient veins.

## Introduction

Severe traumatic lower extremity injuries involving open fractures (Gustilo type IIIB and IIIC) often require free flap coverage for limb salvage. Although microsurgical techniques are more advanced than ever before, these injuries can be challenging to reconstruct because of a higher rate of complications, including flap failure, than in any other anatomical region [1]. Multiple factors impact these complications, and whereas individual factors such as the degree of traumatic injuries and zone of injury cannot be controlled, procedural factors may offer areas for potential improvement, particularly the identification of healthy, reliable recipient vessels and the methods and types of anastomoses formed between recipient and donor vessels. Advances in diagnostic imaging systems, such as Doppler ultrasound examination, computed tomography angiography (CTA), and digital subtraction angiography, have greatly facilitated the identification of appropriate recipient arteries in the lower extremity. Although preoperative diagnostic arterial imaging may be prevalent, preoperative venous evaluation for the lower extremity has not been commonly performed and lacks a well-established diagnostic system. Thus, venous thrombosis has been shown to be the most frequent cause of free flap failure in the lower extremity [1].

Venous size mismatch was shown to be a high-risk factor in flap complications in end-to-end (ETE) venous anastomoses during lower extremity reconstruction [2,3], and the deep venous system has been more reliable as a recipient site for outflow compared with the superficial venous system [4]. Furthermore, end-to-side (ETS) venous anastomoses have been shown to be safe with a low rate of microvascular complications in lower extremity reconstructions [5,6]. However, there is some controversy over whether one- or two-vein anastomoses offer optimal venous outflow in these reconstructions [3,4,7-9]. Heterogeneity in previous studies is attributable to the inclusion of various disorders such as oncologic and traumatic defects. In addition, some bias may exist due to a number of previous studies in this research area arising from the same institution. Thus, the exact roles of anastomotic venous factors, such as venous anastomosis (ETE or ETS), venous outflow (one or two veins), and recipient venous selection (deep or superficial veins), remain unclear.

These venous factors may be crucial for the success of free flap reconstruction of traumatic lower extremity injuries. Therefore, this study aims to investigate factors contributing to microvascular complications in lower extremity Gustilo type IIIB/IIIC injuries reconstructed by free flap with a focus on the three venous factors mentioned above.

## Materials and methods

Study design

This retrospective study was performed in two similar-sized hospitals (Hiroshima Prefectural Hospital and Hiroshima University Hospital) by two senior surgeons (KS and RS) and included patients treated for traumatic lower extremity injuries by free tissue transfers from 2015 to 2020. The population under study was considered unbiased because most patients that required traumatic lower extremity reconstruction in our area were transported by ambulance to either of these two hospitals.

Inclusion and exclusion criteria

Patients with traumatic wounds other than Gustilo type IIIB and IIIC open fractures and with short-term follow-up (<3 months) were excluded from the study. Three patients underwent secondary free flap reconstruction due to initial flap failure. These three secondary free flap reconstructions were included in this study because the time interval between the first and second reconstructions was short and traumatic wounds did not progress to infections including osteomyelitis.

Data collection

A total of 41 patients with 44 free tissue transfers performed for lower extremity reconstruction were identified. All patients were treated according to the same protocol: anterolateral thigh (ALT) flaps were the first choice to repair soft tissue defects, latissimus dorsi musculocutaneous (LDM) flaps with meshed skin graft were used for larger defects, and osteocutaneous flaps such as fibula osteocutaneous flaps were used for soft tissue and bone defects. The recipient vessels were evaluated using CTA, and the presence or absence of deep venous thrombosis (DVT) was examined using Doppler ultrasound preoperatively. All anastomoses were hand sutured, and the patients were administered low-molecular-weight heparin and prostaglandin E1 infusions postoperatively. The following variables were extracted from the included patient data: patient demographics (age and gender), Gustilo type, time from injury to coverage (e.g., <7 days, 7-90 days, or >90 days), lower extremity zone of injury (e.g., thigh, proximal leg, middle leg, distal leg, or foot and ankle), flap type (e.g., fasciocutaneous, musculocutaneous, or osteocutaneous flap), arterial anastomosis (e.g., ETE, flow-through, or ETS), venous anastomosis (e.g., ETE or ETS), venous outflow (e.g., one or two veins), recipient veins (e.g., deep, superficial, or deep and superficial veins), and complications (e.g., partial flap failure, complete flap failure, infection, foot ischemia, or take-back). Partial flap failure was defined as partial necrosis, which required an additional surgical procedure as no conservative nonsurgical treatment is currently available.

Statistical analysis

These data were analyzed using Fisher’s exact test or one-way analysis of variance. When the subject number in any category was more than five, two-tailed Student’s t-test was applied. Statistical significance was defined as p < 0.05.

Surgical technique

**Figure 1:**
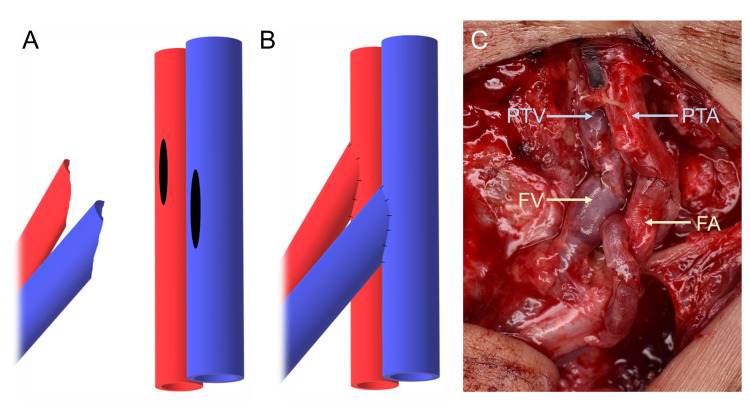
Y-shaped end-to-side (ETS) anastomosis of both the artery and vein. (A) Schema of pre-anastomosis. (B) Schema of post-anastomosis. (C) Photograph of post-anastomosis. PTA: posterior tibial artery; PTV: posterior tibial vein; FA: flap artery; FV: flap vein.

ETS anastomosis was performed as follows: the recipient vein was first clamped, and an elliptical venotomy was performed using microscissors. The length of the elliptical aperture was set to twice the donor venous diameter, an oblique excision was then made in the donor vein using microscissors to broaden the circumferences, and Y-shaped ETS venous anastomosis was performed. Subsequently, Y-shaped ETS arterial anastomosis was performed in a similar fashion to that in which arteriotomy is usually performed using a 1-mm biopsy punch (Figure 1) [10].

## Results

The average patient age was 52 years, with the majority (75.6%) being male, and the majority of Gustilo type injuries were IIIB (n = 41, 93.2%) (Table 1).

**Table 1:** Patient demographics and operative details. ETE: end-to-end; ETS: end-to-side.

Characteristic		Value (%)
Age		52 ± 21
Gender	Male	31 (75.6)
	Female	10 (24.4)
Gustilo type	IIIB	41 (93.2)
	IIIC	3 (6.8)
Time from injury to coverage	<7 days	9 (20.5)
	7-90 days	31 (70.5)
	>90 days	4 (9.1)
Lower extremity zone of injury	Thigh	1 (2.3)
	Proximal leg	5 (11.4)
	Middle leg	15 (34.1)
	Distal leg	15 (34.1)
	Foot and ankle	8 (18.2)
Flap type	Fasciocutaneous	24 (54.5)
	Musculocutaneous	8 (18.2)
	Osteocutaneous	12 (27.3)
Arterial anastomosis	ETE	25 (56.8)
	Flow-through	5 (11.4)
	ETS	14 (31.8)
Venous anastomosis	ETE	28 (63.6)
	ETS	16 (36.4)
Venous outflow	One vein	20 (45.5)
	Two veins	24 (54.5)
Recipient venous selection	Deep vein	26 (59.1)
	Superficial vein	13 (29.5)
	Deep and superficial vein	5 (11.4)
Complications	Any flap failures	8 (18.2)
	Partial flap failure	3 (6.8)
	Second free flap	1 (2.3)
	Complete flap failure	5 (11.4)
	Amputation	2 (4.5)
	Second free flap	2 (4.5)
	Infection	2 (4.5)
	Foot ischemia	1 (2.3)
Microvascular complications	Take-back (due to venous thrombosis)	8 (18.2)
	Salvage	3 (37.5)

**Figure 2:**
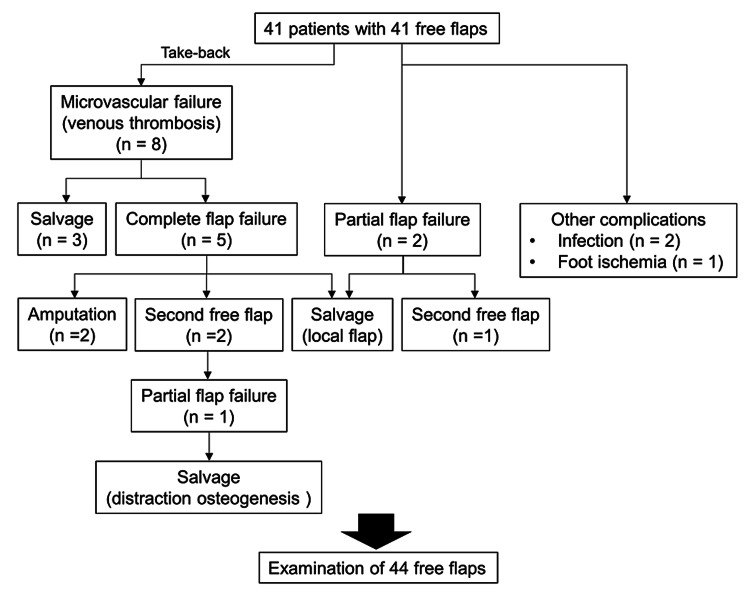
Study flowchart.

The breakdown of complications was as follows: partial flap failure (n = 3, 6.8%), complete flap failure (n = 5, 11.4%), infection (n = 2, 4.5%), and foot ischemia (n = 1, 2.3%). The patients with complete flap failures ultimately resulted in amputations (n = 2) or second free flaps (n = 2). Eight patients returned to the operating room due to venous thrombosis (n = 8, 18.2%); three of these patients’ limbs were salvaged by vein graft transfer, whereas five patients experienced complete flap failure (Figure 2).

The following factors are believed to have played a role in venous thrombosis: large-to-small (>1 mm size mismatch) venous anastomosis (n = 2) and recipient vein insufficiency possibly due to post-traumatic vessel disease (PTVD) (n = 6). It was suspected that the failure of three partial flaps was because of the large size of the flaps rather than any microvascular failure. Venous thrombosis is closely related to complete flap failure and has devastating consequences for patients with lower limb injuries. We, therefore, examined our stratified data to assess the risk factor of complications (Table 2) of three venous factors: anastomosis, outflow, and selection.

**Table 2:** Stratified analyses of complication rates. ETE: end-to-end; ETS: end-to-side.

	Venous thrombosis		Complete flap failure		Any flap failures		Overall complications	
	Number (%)	p value	Number (%)	p value	Number (%)	p value	Number (%)	p value
Total	8 (18.2)		5 (11.4)		8 (18.2)		11 (25)	
Gustilo type								
IIIB	8 (19.5)	1	5 (12.2)	1	8 (19.5)	1	11 (26.8)	0.56
IIIC	0 (0)		0 (0)		0 (0)		0 (0)	
Time from injury to coverage								
<7 days	0 (0)	0.13	0 (0)	0.32	0 (0)	0.30	1 (11.1)	0.57
7-90 days	8 (25.8)		5 (16.1)		7 (22.6)		9 (29)	
>90 days	0 (0)		0 (0)		1 (25)		1 (25)	
Lower extremity zone of injury								
Thigh	0 (0)	0.40	0 (0)	0.27	0 (0)	0.29	0 (0)	0.17
Proximal leg	0 (0)		0 (0)		0 (0)		0 (0)	
Middle leg	4 (26.7)		1 (6.7)		3 (20)		5 (33.3)	
Distal leg	4 (26.7)		4 (26.7)		5 (33.3)		6 (40)	
Foot and ankle	0 (0)		0 (0)		0 (0)		0 (0)	
Flap type								
Fasciocutaneous	3 (12.5)	0.58	1 (4.2)	0.23	1 (4.2)	0.01	3 (12.5)	0.08
Musculocutaneous	2 (25)		2 (25)		4 (50)		4 (50)	
Osteocutaneous	3 (25)		2 (16.7)		3 (25)		4 (33.3)	
Arterial anastomosis								
ETE	6 (24)	0.44	3 (12)	0.74	5 (20)	0.91	8 (28)	0.47
Flow-through	1 (20)		1 (20)		1 (20)		1 (20)	
ETS	1 (7.1)		1 (7.1)		2 (14.3)		2 (16.7)	
Venous anastomosis								
ETE	7 (25)	0.22	4 (14.3)	0.64	6 (21.4)	0.69	8 (28.6)	0.71
ETS	1 (6.3)		1 (6.3)		2 (12.5)		3 (18.8)	
Venous outflow								
One vein	6 (30)	0.07	3 (15)	0.65	4 (20)	1	6 (30)	0.51
Two veins	2 (8.3)		2 (8.3)		4 (16.7)		5 (20.8)	
Recipient venous selection								
Deep vein	2 (7.7)	0.06	2 (7.7)	0.65	5 (19.2)	0.95	6 (23.1)	0.84
Superficial vein	5 (38.5)		2 (15.4)		2 (15.4)		4 (30.8)	
Deep and superficial veins	1 (20)		1 (20)		1 (20)		1 (20)	

Venous anastomosis

ETE venous anastomoses were more commonly used (n = 28, 63.6%) than ETS anastomoses (n = 16, 36.4%). Although the venous thrombosis rates of ETS anastomoses were lower than those of ETE anastomoses (6.3% versus 25%), the difference was not statistically significant (p = 0.22). Furthermore, no association was found between the differences in venous anastomosis type and total flap failure (p = 0.64), any flap failures (p = 0.69), or overall complications (p = 0.71).

Venous outflow

The number of flaps was compared between one-vein and two-vein groups (n = 20 versus n = 24). Although the venous thrombosis rates of the one-vein group were higher than those of the two-vein group (30% versus 8.3%), this difference did not reach statistical significance (p = 0.07). Moreover, no association could be found between the differences in the venous outflow and total flap failure (p = 0.65), any flap failures (p = 1), or overall complications (p = 0.51).

Recipient venous selection

Deep veins were most commonly selected (n = 26, 59.1%). The venous thrombosis rates of the deep vein group were lowest (7.7%), whereas those of the superficial vein group were highest (38.5%) among the three groups; however, this difference did not reach statistical significance (p = 0.06). In addition, there was no association between the differences in the recipient venous selection and total flap failure (p = 0.65), any flap failures (p = 0.95), or overall complications (p = 0.84).

Confounding factors and biased distribution in three venous factors

Although DVT is considered a risk factor that compounds the impact of anastomotic venous factors the most, all patients were free of DVT in the preoperative examination. We examined other confounding factors for venous thromboses, such as the mean time from injury to coverage (7-90 days versus <7 days and >90 days), lower extremity zone of injury (middle and distal legs versus thigh, proximal leg, foot, and ankle), and flap type (musculocutaneous and osteocutaneous versus fasciocutaneous) (Table 3).

**Table 3:** Confounding factors for the occurrence of venous thrombosis. OR: odds ratio; CI: confidence interval; DVT: deep venous thrombosis.

	Venous thrombosis (n = 8)	No venous thrombosis (n = 36)	OR (95% CI)	p value
DVT	0	0		-
Mean times from injury to coverage (days)	39.4	34.4		0.79
	Number (%)	Number (%)		
Time from injury to coverage				
7-90 days	8 (25.8)	23 (74.2)	9.77 (0.5–182.8)	0.08
<7 days and >90 days	0 (0)	13 (100)		
Lower extremity zone of injury				
Middle and distal legs	8 (26.7)	22 (73.3)	10.96 (0.6–204.7)	0.04
Thigh, proximal leg, and foot and ankle	0 (0)	14 (100)		
Flap type				
Musculocutaneous and osteocutaneous	5 (25)	15 (75)		
Fasciocutaneous	3 (12.5)	21 (87.5)	0.43 (0.1–2.1)	0.44

To elucidate whether the risk factors of venous thrombosis in three venous factors were biased, we compared the breakdown of venous thrombosis in three venous factors with two suspected high-risk factors: time from injury to coverage (7-90 days, p = 0.08) and high-risk zone of the lower extremity (middle and distal legs, p = 0.04) (Table 4).

**Table 4:** Breakdown of three venous factors with confounding high-risk factors. ETE: end-to-end; ETS: end-to-side. *High-risk zone means middle and distal legs.

	Venous thrombosis rate (%)	Proportions of 7–90 days from injury to coverage (%)	p value	Proportions of high-risk zone* (%)	p value
Venous anastomosis					
ETE	25	24 (85.7)	0.01	19 (67.9)	1
ETS	6.3	7 (43.8)		11 (68.8)	
Venous outflow					
One vein	30	15 (75)	0.74	13 (65)	1
Two veins	8.3	16 (66.7)		17 (70.8)	
Recipient venous selection					
Deep vein	7.7	16 (61.5)	0.31	16 (61.5)	0.33
Superficial vein	38.5	11 (84.6)		11 (84.6)	
Deep and superficial veins	20	4 (80)		3 (60)	

There was significant bias in the venous anastomosis for time from injury to coverage (p = 0.01) but no significant bias in the high-risk zone of the lower extremity (p = 1). It was shown that the venous outflow has no bias in the two suspected high-risk factors (p = 0.74 and p = 1, respectively). Moreover, there was no significant bias in recipient venous selection with the two suspected high-risk factors (p = 0.31 and p = 0.33, respectively).

## Discussion

Venous thrombosis caused by congestion has been shown to be the most common cause of flap failure [1]. Our results confirmed that the cause of total flap failure was entirely attributable to venous thrombosis, and the most common cause of venous thrombosis was thought to be PTVD (n = 6, 75%). PTVD can result from changes in the vessel walls and the perivascular tissues and lead to postoperative thrombosis [4,11]. Recent studies have shown that lower extremity venous duplex ultrasound imaging before free flap transfer is a useful tool [12]; however, it may be difficult to detect microscopic venous abnormality such as early PTVD. Although multiple different factors can be associated with the flap complications of traumatic lower extremity reconstruction, it should be noted that procedural measures, which take venous factors into account and aim to reduce venous thrombosis, play a major role in reducing the risk of flap failure. Our results showed that three venous factors had some impact on venous thrombosis rates, albeit only a limited impact on other complications. These findings were considered to be significant for the salvage of venous thrombosis (n = 3) and other complications irrespective of venous thrombosis.

Venous anastomosis

Our results demonstrated that the venous thrombosis rates of ETS anastomoses were lower than those of ETE anastomoses (6.3% versus 25.0%, p = 0.22); however, there was significant bias in time from injury to coverage (p = 0.01). Notably, the ETS anastomosis group had a lower proportion of cases within 7-90 days from injury to coverage than ETE anastomosis groups, suggesting that ETS anastomosis groups were selected in comparatively advantageous conditions. Although ETS venous anastomoses have been mainly reported in the head and neck region [13], there have been several successful reports in the lower extremity [5,6]. In addition, flow-through venous anastomosis has also been shown to have a lower rate of microvascular complications than ETE anastomosis in oncologic lower extremity reconstruction [14]. The reason for the superiority of ETS or flow-through venous anastomosis is thought to be the preservation of distal pump effects. These pump effects promote venous return, washing out the venous blood around the anastomotic site; accordingly, the risk of venous thrombosis is reduced by the prevention of venostasis [14]. ETS anastomosis is superior to flow-through anastomosis when there is a vessel size mismatch between recipient and donor vessels. Miyamoto et al. reported that large-to-small ETS venous anastomosis can be a breakthrough option when only a small recipient vein is available [6]. We used Y-shaped ETS anastomosis because Y-shaped ETS venous anastomosis can theoretically prevent anastomotic complications by broadening anastomotic diameter; however, one flap using this method resulted in venous thrombosis, possibly due to PTVD. ETS venous anastomosis is more burdensome but may be more favorable than ETE anastomosis; particularly, ETS venous anastomosis can be an alternative option when there is a vein size mismatch.

Venous outflow

There have been conflicting reports on whether one- or two-vein anastomoses are optimal in lower extremity flap reconstructions. Several studies showed that two-vein anastomoses reduced complication rates compared with one-vein anastomoses [3,7]. Our results indicated that the venous thrombosis rates of two-vein anastomoses were lower than those of one-vein anastomoses (8.3% versus 30%, p = 0.07), and there was little biased distribution of high-risk factors between both groups. Our findings are corroborated by a previous report confined to traumatic lower extremity injuries [3]. However, some previous studies have reported that the number of veins involved in anastomosis did not impact postoperative complications [8,9] and that one-vein anastomosis flaps were related to fewer total flap failures in comparison with two-vein anastomosis flaps [4]. Traumatic lower extremity injuries often result in PTVD, which leads to an increase in venous pressure. PTVD has a risk of reducing venous blood velocity, which may increase the risk of venostasis and thrombosis. Thus, two-vein anastomoses may provide additional drainage and offer a beneficial backup system when one vein becomes compromised.

Recipient venous selection

In the lower extremity reconstruction, few studies have investigated whether deep or superficial venous systems are more reliable. Often, the selection of deep or superficial systems depends on the clinical scenario and anatomical region of the injury. We selected deep venous systems most frequently (59.1%) on the basis of the convenience of their location (i.e., the recipient vein was close to the recipient artery). Our results showed that the venous thrombosis rates in the deep vein group were lowest (7.7%), whereas those of the superficial vein group were the highest (38.5%) among the three groups. Our findings are corroborated by a previous report by Lorenzo et al. [4]. The superficial venous system is often far from the recipient artery and is more frequently damaged due to PTVD [4]; however, it is easy to approach and offers a more constant vein size. Thus, the superficial venous system may be the preferred option when there is a vein size mismatch between donor and recipient deep veins.

Limitations

There were several limitations in this study. First, the sample size of the study was small; therefore, further prospective studies with larger sample sizes are required to fully assess the effects of the three venous factors. Second, we examined bias by comparing the effects of the three venous factors with time from injury to coverage and the high-risk zone of the lower extremity. However, there might be other confounding risk factors, particularly unknown venous abnormality, such as venous reflux, which was unverified in our study. Third, the postoperative venous outflow from donor to recipient vein and, in particular, how two-vein anastomoses contributed to outflow remain unknown due to the lack of postoperative venous imaging.

## Conclusions

In the free flap reconstruction of traumatic lower extremity injuries, identifying healthy reliable recipient veins is a major priority to prevent microvascular complications. It is therefore desirable to further develop reliable venous imaging systems. Nevertheless, two-vein ETS anastomoses to deep recipient veins are key venous factors in the prevention of venous thrombosis. ETS venous anastomosis may be more reliable than ETE anastomosis. In particular, ETS venous anastomosis can be an alternative option in cases with a vein size mismatch. 
